# Dietary Supplementation of *Astragalus membranaceus* Extract Affects Growth Performance, Antioxidant Capacity, Immune Response, and Energy Metabolism of Largemouth Bass (*Micropterus salmoides*)

**DOI:** 10.1155/2024/3893671

**Published:** 2024-03-01

**Authors:** Xuanshu He, Anqi Chen, Zhihong Liao, Jian Zhong, Anda Cheng, Xinghua Xue, Fuyuan Li, Mengdie Chen, Rong Yao, Wei Zhao, Jin Niu

**Affiliations:** ^1^State Key Laboratory of Biocontrol, Guangdong Provincial Key Laboratory for Aquatic Economic Animals and Southern Marine Science and Engineering Guangdong Laboratory (Zhuhai), School of Life Sciences, Sun Yat-sen University, Guangzhou, China; ^2^Zhanjiang Customs, Zhanjiang, China; ^3^Beijing Centre Biology Co. Ltd., Beijing, China

## Abstract

The present study investigated the effects of *Astragalus membranaceus* extract (AME) on growth performance, immune response, and energy metabolism of juvenile largemouth bass (*Micropterus salmoides*). Seven diets containing 0%, 0.1%, 0.2%, 0.3%, 0.4%, 0.5%, and 0.6% AME (Con, AME0.1, AME0.2, AME0.3, AME0.4, AME0.5, and AME0.6 groups) were formulated and fed to *M. salmoides* for 8 weeks. Final body weight (FBW), feed intake (FI), weight gain (WG), and specific growth rate (SGR) were all significantly higher in AME0.4 group than in Con group (*P* < 0.05). Feed conversion rate (FCR) was significantly improved in AME0.5 group compared with Con group (*P* < 0.05). Whole-body crude protein contents were significantly increased in AME0.2 group (*P* < 0.05). Whole-body crude lipid contents were significantly lower in AME0.2 and AME0.3 groups, while muscle lipid was upregulated by dietary AME (*P* < 0.05). Hepatic malondialdehyde (MDA) contents were significantly lowered in AME0.3 and AME0.4 groups, and catalase (CAT) activities were significantly increased in AME0.1 and AME0.2 groups (*P* < 0.05). Plasma aspartate aminotransferase (AST) level was significantly lowered in AME0.5, and AME0.6 groups, and alanine aminotransferase (ALT) level was lowered in AME0.5 groups (*P* < 0.05). Plasma triglyceride was declined in AME0.6 group, and glucose was decreased by 0.3%−0.5% AME (*P* < 0.05). Significantly higher hepatocyte diameter, lamina propria width, and submucosal layer thickness were recorded in AME0.6 groups, while the longest villi height was obtained in AME0.2 and AME0.3 groups (*P* < 0.05). The mRNA expression levels of insulin-like growth factor 1 (*igf1*) revealed the growth-promoting effect of AME. The anti-inflammatory and antiapoptotic effects of AME were demonstrated by transcription levels of interleukin 8 (*il-8*), tumor necrosis factor-alpha (*tnf-a*), caspase, B-cell lymphoma-xl (*Bcl-xl*), bcl-2 associated x (*Bax*), and bcl-2-associated death protein (*Bad*). The transcription levels of lipid metabolism and gluconeogenesis related genes, including acetyl-CoA carboxylase alpha (*acc1*), fatty acid synthase (*fasn*), fatty acid binding protein 1 (*fabp1*), phosphoenolpyruvate carboxykinase 2 (*pepck2*), and glucose-6-phosphatase catalytic subunit 1a (*g6pc*), were reduced by AME treatment, while the levels of glycolysis-related genes, including glucokinase (*gck*) and pyruvate kinase (*pk*), were the highest in AME0.2 and AME0.3 groups (*P* < 0.05). According to polynomial regression analysis of SGR, WG, FCR, whole-body crude lipid, MDA, and ALT, the optimal AME supplementation level was estimated to be 0.320%−0.429% of the diet. These results provided insights into the roles of AME in regulating immunity and metabolism, which highly indicated its potential as immunostimulants and metabolic regulators in diverse aquatic animals.

## 1. Introduction

The aquaculture industry kept flourishing in the past few decades for the increasing dietary needs of consumers. Largemouth bass (*Micropterus salmoides*), a carnivorous freshwater economic fish, is massively reared in south China because of its high nutritional value, fast growth rate, and strong market demand [1]. However, problems have occurred as high-density rearing continuously existed. Frequent outbursts of infectious diseases caused by aquatic pathogens strongly limited the farming industry of *M. salmoides* [2, 3], bringing serious economic losses to the fishery and impeding the sustainable development of aquaculture [4]. Therefore, preventive and therapeutic methods are highly required to counteract pathogen infections and subsequent diseases. Antibiotics used to be primary solutions to preventing and treating disease outbreaks [5], whereas antibiotic abusing inevitably caused chemical accumulation in aquatic environments and developed antibiotic-resistant bacterial strains, which led to environmental pollution and eventually threatened the health of animals and even humans [6, 7]. Therefore, it is of great necessity to replace antibiotics with more natural compounds for less side effects in aquatic animal husbandry. Herbs and herbal extracts thus started appealing to researchers as proper alternatives for being much safer and more environmental-friendly. In recent years, there has been an increasing interest in the research of the immunomodulatory effects of herbal extract on *M. salmoides*. It has been discovered that lots of herbal products such as sweet wormwood (*Artemisia annua*) extract [8], hardy rubber tree (*Eucommia ulmoides* Oliver) extract [9], mulberry leaf (*Morus alba*) extract [10] and Yinchenhao (*Artemisiae Scopariae*) decoction [11] positively regulated liver antioxidant capacities and mitigated impairments of immunity in *M. salmoides*, displaying the potential of herbal products as dietary immunopotentiators.

As reported in various species, herbal medicine showed considerable efficacy in promoting growth performance, augmenting immunity, and enhancing disease resistance [7, 12], which could be attributed to its active substances, including polysaccharides, flavonoids, alkaloids, polyphenols, and so on [13]. Among the great number of medicinal plants, *Astragalus membranaceus*, also known as Huangqi in Chinese, a kind of traditional herbal plant, has long been regarded as an important agent for Chinese medicine in history. It has been proven in numerous researches that *A. membranaceus* showed capabilities in enhancing antioxidant activities [14], attenuating inflammation stress [15, 16], affecting autophagy responses [17], regulating lipid metabolism [18], and treating diabetes mellitus [19] in humans and other mammals. Particularly, *Astragalus* polysaccharides, one of the most crucial effective constituents in *A. membranaceus*, showed its great therapeutic potential as immunomodulatory and antitumor agents in multiple organisms [20–22]. Flavonoids in Genus *Astragalus*, as well as other herbal plants, have also been revealed to be potent in exerting antioxidant and anti-inflammatory activities [23–25], showing anticancer effects [26] and potential antimicrobial agents [27]. In the past few years, multiple studies have illustrated the functions of *A. membranaceus* and its active extracts in regulating growth performance, immunity, antioxidant capacity, gut health, apoptosis, and disease resistance against pathogens in diverse aquatic species, including zebrafish *Danio rerio* [28], Asian seabass *Lates calcarifer* [29], turbot *Scophthalmus maximus* [30], common carp *Cyprinus carpio* [31], Nile tilapia *Oreochromis niloticus* [32], snakehead *Channa argus* [33], and Pacific white shrimp *Litopenaeus vannamei* [34, 35], which portrayed promising future of the popularization of *A. membranaceus* and its active extracts in aquaculture industry against pathogens and environmental stress.

As far as we know, the effects of *A. membranaceus* on *M. salmoides* have yet to be fully illustrated. Considering the diverse regulatory effects of *A. membranaceus* on other aquatic species, it would be meaningful to elaborate on whether the observation of such effects could be extended to *M. salmoides*. Therefore, we attempted to elucidate the potential effects of *A. membranaceus* on regulating growth, antioxidant status, immunity, hepatic and intestinal histology, as well as energy metabolism in *M. salmoides*. The active components of *A. membranaceus* extract used in this study were mainly polysaccharides and flavonoids.

## 2. Materials and Methods

### 2.1. Experimental Diets

Seven isonitrogenous and isolipidic diets were designed and formulated according to Table 1. Apart from basal diet for Con group, 0.1%, 0.2%, 0.3%, 0.4%, 0.5%, and 0.6% *A. membranaceus* extract (AME) powder was, respectively, supplemented in the basal diet to form six experimental diets and respectively assigned to AME0.1, AME0.2, AME0.3, AME0.4, AME0.5, and AME0.6 groups. Cellulose microcrystalline was used for eliminating the difference in quantity caused by unequal supplementation of AME powder. The AME powder utilized in the present study was kindly provided by Beijing Centre Biology Co., Ltd. and was analyzed to contain 3.50% polysaccharide and 0.152% flavonoids as active components and 4.75% protein. Overall, 50% cellulose were also included in the AME powder as herbal drug carriers. The procedure of preparing the AME powder is presented in *Supplementary 1*. White fish meal, soybean meal, wheat gluten, beer yeast, and krill meal were used as main protein sources. Fish oil and soybean lecithin were used as main lipid sources. For the preparation of diets, all the solid ingredients were ground to fine particles and sieved by a 320 *μ*m mesh. A moist dough was obtained by blending the ingredients with the oils and mixing with water (0.4 L kg^−1^) thoroughly in a commercial mixer (A-200T Mixer Bench Model unit, Resell Food Equipment Ltd., Ottawa, Canada). About 2.0 mm pellets were obtained by having the dough extruded at 80°C and pelletized using a twin-screw extruding machine (VALVA60-III, Value Machinery and Equipment Co., Ltd., Guangzhou, China) and then dried at 16°C until constant weight. All the diets were stored at −20°C for fish rearing.

### 2.2. Fish and Experimental Conditions

Juvenile largemouth bass were purchased from Shunye Fishery Company (Foshan, China) and then acclimated in an indoor circulation system at Sun Yat-sen University with feeding a commercial diet (crude protein: 45%, crude lipid: 10%; Tongwei Co., Ltd., China) for 2 weeks. A total of 420 healthy juveniles of similar size (initial body weight: 10.09 ± 0.02 g) were fasted for 24 hr and then randomly allocated to 21 cement tanks of 180 L at a density of 20 fish per tank. Every diet was assigned to three parallel tanks. Fish were hand-fed to apparent satiation at 9:00 and 16:00 every day for 56 days. Filtered water was supplied to each tank at the flow rate of 9 L min^−1^. Used water was discarded, and freshwater was replenished in the tanks every week. During the whole feeding trial, water temperature, pH, and dissolved oxygen concentration were maintained at 26.0 ± 2.0°C, 8.0 ± 0.2, and over 6.0 mg L^−1^. Ammonia nitrogen concentration was controlled at less than 0.2 mg L^−1^ for the whole period.

### 2.3. Growth Performance and Morphology

At the termination of the feeding trial, the experimental fish were fasted for 24 hr before sampling. The amount of feed intake of each tank was calculated, and the number and total weight of fish in each tank were determined for the calculation of growth performance-related parameters. Four fish from each tank were anesthetized using 300 mg L^−1^ MS-222 (Sigma, St. Louis, USA) before measuring the body length, body weight, visceral mass weight, and hepatic weight of each fish, which were then used for the analysis of morphological parameters.

### 2.4. Proximate Composition Analysis of Whole Body, Muscle, and Feed

Three fish were randomly selected from each tank for analysis of proximate composition in the whole body. The flesh of four fish was isolated from each tank for determination of proximate composition in muscle. The analysis of crude protein, crude lipid, moisture, and ash in the whole body, muscle, and feed was conducted according to the guidance of the Association of Official Analytical Chemists [36]. Crude protein contents were determined by the Dumas combustion method (Dumas nitrogen analyzer, N pro (DT Ar/He Basic), Gerhardt GMBH & Co. KG, Germany). Crude lipid contents were detected by the Soxhlet extraction method (Soxtec System HT6, Tecator AB, Höganäs, Sweden). Moisture contents were determined by drying samples at 105°C until constant weight. Ash contents were calculated by burning samples in a muffle furnace (M110, Thermo Scientific, Waltham, USA) over 550°C till complete carbonization.

### 2.5. Antioxidant Capacity Analysis

Liver samples were rapidly dissected and placed in liquid nitrogen for analysis of antioxidant capacity. For every tank, liver lysate was prepared by homogenizing liver samples pooled from three fish in nine volumes (1 : 9, w/v) of ice-cold phosphate-buffered saline (PBS, pH 7.4) and collecting the supernatants after centrifuging the lysate at 3, 500 rpm for 10 min at 4C. The activities of superoxide dismutase (SOD), glutathione peroxidase (GSH-PX), catalase (CAT), and the contents of malondialdehyde (MDA) were determined using reagent kits under the guidance of manufactures' instructions (Nanjing Jiancheng Bioengineering Institute, Nanjing, China). To be specific, xanthine oxidase method was used for SOD activity detection. Superoxide anion, which originated from xanthine, formed nitrite by oxidizing hydroxylamine, and SOD activity was signified by the degree of inhibition of such oxidization. GSH-PX activity was reflected by the consumption of glutathione, the substrate of the enzymatic reaction, which turned hydrogen peroxide (H_2_O_2_) into water. CAT activity was determined by the ammonium molybdate method. Ammonium molybdate terminated the breakdown of H_2_O_2_ catalyzed by CAT and complexed with the residual H_2_O_2_, and CAT activity was then characterized by the amounts of the complexation product. MDA contents were determined by monitoring its condensation level with thiobarbituric acid. The tissue protein concentrations were detected following the Bradford method for expressing the enzymatic activities with the unit of U mg^−1^ protein. All the parameters were detected by UV–Vis spectrophotometer (Shimadzu UV-2450, Kyoto, Japan).

### 2.6. Blood Collection and Plasma Biological Analysis

Blood of four fish in each tank was collected from the caudal vein using 1 mL sterile syringes precoated with heparin sodium and centrifuged at 5,000 rpm for 10 min at 4°C. Plasma samples were aspirated from the upper layer and pooled before being stored at −80°C for future analysis. Plasma biological parameters, including aspartate aminotransferase (AST), alanine aminotransferase (ALT), alkaline phosphatase (AKP), acid phosphatase (ACP), triglyceride (TG), total-cholesterol (T-CHO), and glucose (GLU), were detected in 96-well microplates using corresponding reagent kits (Nanjing Jiancheng Bioengineering Institute, Nanjing, China). AST and ALT were measured using the Reitman–Frankel method. AKP and ACP were measured using the phendisodium phosphate colorimetric method. TG, T-CHO, and GLU were detected by glycerol-3-phosphate oxidase–peroxidase chromogenic method, cholesterol oxidase-peroxidase chromogenic method, and glucose oxidase–peroxidase chromogenic method, respectively [37]. The changes in absorbance were monitored by an ultra-micro full-wavelength absorption light microplate reader (Epoch, BioTek Instruments, Inc., Winooski, USA).

### 2.7. Hepatic and Intestinal Histology

After blood sampling, fish were dissected immediately, and liver and midgut samples were both separated and fixed in 4% paraformaldehyde for 24 hr. The histological sections were made according to the method described by Yin et al. [38] with some modifications. To be specific, both hepatic and intestinal tissues were dehydrated in graded ethanol before being embedded into paraffin and made into tissue sections of 4 *µ*m. The sections were then stained with hematoxylin and eosin, observed, and photographed using an upright microscope (Eclipse Ni-E, Nikon, Japan). ImageJ software (National Institutes of Health, Bethesda, USA) was utilized for the measurement of hepatic cell diameter, villus height, villus width, lamina propria width, muscular layer thickness, and submucosal layer thickness.

### 2.8. Total RNA Extraction and Gene Expression Analysis

For every tank, liver tissues from three fish were pooled as one sample for the detection of target gene transcription levels. Hepatic total RNA was extracted using RNAeasy™ Animal RNA isolation kit (Beyotime Biotechnology, Shanghai, China), and the integrity of RNA was evaluated by 1.2% agarose gel electrophoresis. Meanwhile, the concentration of the RNA extract was analyzed by a nanodrop spectrophotometer (Nanodrop 2000, Thermo Scientific, USA). cDNA was prepared by a two-step reaction, including the removal of genomic DNA and reverse transcription, using an *Evo M-MLV* Reverse transcription reagent kit (Accurate Biology, Hunan, China). LightCycler 480 Ⅱ quantitative real-time system (Roche Diagnostics, Basel, Switzerland) was utilized to operate quantitative real-time PCR (qRT-PCR), which was performed in a 10 *μ*L reaction system consisting of 5 *μ*L of 2 × SYBR Green *Pro Taq* HS Premix (SYBR Green *Pro Taq* HS Premix qPCR reagent kit, Accurate Biology, Hunan, China), 0.2 *μ*L of each primer, 2.6 *μ*L diethyl pyrocarbonate (DEPC) water and 2 *μ*L of cDNA diluent (cDNA product: DEPC water = 1 : 9). The qRT-PCR reactions were conducted as follows: the step of preincubation for 10 min at 95°C, 40 amplification cycles of denaturation for 5 s at 95°C, annealing for 30 s at 60°C and extension for 30 s at 72°C, and the validation of reaction quality by running standard melting curves. The relative quantification of the expressions of target genes was analyzed by 2^-*ΔΔ*Ct^ method [39]. The sequence information of primer pairs for detection of all target genes and *ef-1a*, the internal control, are shown in *Supplementary 2*.

### 2.9. Statistical Analysis

All results were presented as means ± SEM, which were calculated from three replication tanks. Significant differences between groups were analyzed by one-way analysis of variance (ANOVA) and Tukey's honestly significant difference (Tukey HSD) tests. *P* < 0.05 was defined as statistically different. Orthogonal polynomial contrast analyses were performed to determine whether the effects of AME on various parameters were linear or quadratic. Second-degree polynomial regression analysis was adopted to estimate the optimal supplementation level of AME for *M. salmoides*.

The processing of statistical analysis in this experiment was accomplished by SPSS Statistics 23.0 software (IBM, Chicago, USA).

## 3. Results

### 3.1. Growth Performance, Feed Efficiency, and Morphological Parameters

**Figure 1:**
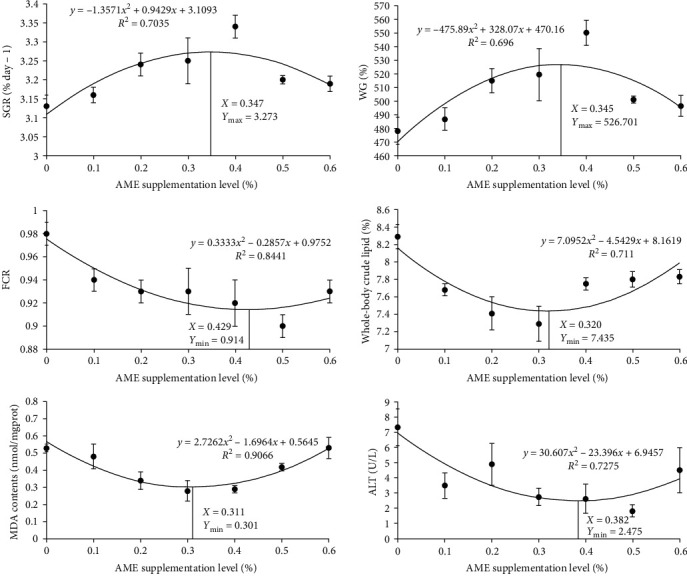
Polynomial regression analysis of SGR, WG, FCR, whole-body crude lipid, MDA, and ALT for *M. salmoides* fed graded dietary AME.

As shown in Table 2, FBW was significantly higher in AME0.4 group than in Con and AME0.1 groups (*P* < 0.05). FI, WG, and SGR were significantly increased in AME0.4 group compared with Con, AME0.5, and AME0.6 groups (*P* < 0.05). SR was not affected by AME treatment (*P* > 0.05). FCR of AME0.5 group was significantly lower than that of Con group (*P* < 0.05), while PER of AME0.4 and AME0.5 groups was significantly higher than that of Con group (*P* < 0.05). The CF of AME0.2, AME0.3, AME0.5, and AME0.6 groups were all significantly higher than that of Con group (*P* < 0.05). VSI was significantly lower in AME0.2 group than in AME0.5 group (*P* < 0.05), and HSI of AME0.2 group was significantly lower than those of AME0.5 and AME0.6 groups (*P* < 0.05). By performing second-degree polynomial regression analysis of SGR, WG, and FCR, the optimal supplementation levels of AME were estimated to be 0.347%, 0.345%, and 0.429% of the diet (Figure 1).

### 3.2. Proximate Composition of Whole-Body and Muscle

As Table 3 presents, the whole-body of AME0.2 groups contained significantly higher crude protein contents than that of Con and AME0.5 groups (*P* < 0.05), while the crude lipid contents were significantly lower in AME0.2 and AME0.3 groups than in Con group (*P* < 0.05). AME0.6 group has significantly higher whole-body ash contents than Con group (*P* < 0.05). As for muscle, the highest crude protein contents were recorded in AME0.3 and AME0.6 groups. The crude lipid contents of AME0.1, AME0.2, AME0.4, and AME0.5 groups were significantly higher than that of Con group (*P* < 0.05), and the crude lipid contents also showed an increasing trend in AME0.3 and AME0.6 groups (*P* > 0.05). Second-degree polynomial regression analysis of whole-body crude lipid contents revealed that the optimal supplementation level of AME was 0.320% of the diet (Figure 1).

### 3.3. Hepatic Antioxidant Ability

The hepatic antioxidant ability-related parameters are shown in Table 4. The MDA contents declined initially and elevated again as the dietary AME level rose, with obtaining the lowest value in AME0.3 group. AME0.1 and AME0.2 groups had significantly higher CAT activities than Con group (*P* < 0.05). No statistical differences were recorded for SOD and GSH-PX activities, although a decreasing trend was shown in GSH-PX activities by AME treatment (*P* > 0.05). The optimal supplementation level of AME was estimated to be 0.311% of the diet by conducting second-degree polynomial regression analysis of MDA contents (Figure 1).

### 3.4. Plasma Biological and Immunological Parameters

As shown in Table 5, the levels of plasma AST and ALT decreased in all AME groups. AST contents were significantly lower in AME0.5 and AME0.6 groups, while ALT contents were significantly lower in AME0.5 group (*P* < 0.05). The levels of AKP and ACP both increased at first and then decreased as the level of dietary AME increased, although no statistical differences were shown (*P* > 0.05). AME supplementation significantly lowered TG contents in AME0.6 groups (*P* < 0.05) while exerting no effects on T-CHO contents among all the groups (*P* > 0.05). AME0.3, AME0.4, and AME0.5 groups had significantly lower GLU contents than Con group (*P* < 0.05). Second-degree polynomial regression analysis of ALT levels showed that the optimal supplementation level of AME was 0.382% of the diet (Figure 1).

### 3.5. Hepatic and Intestinal Histology

**Figure 2:**
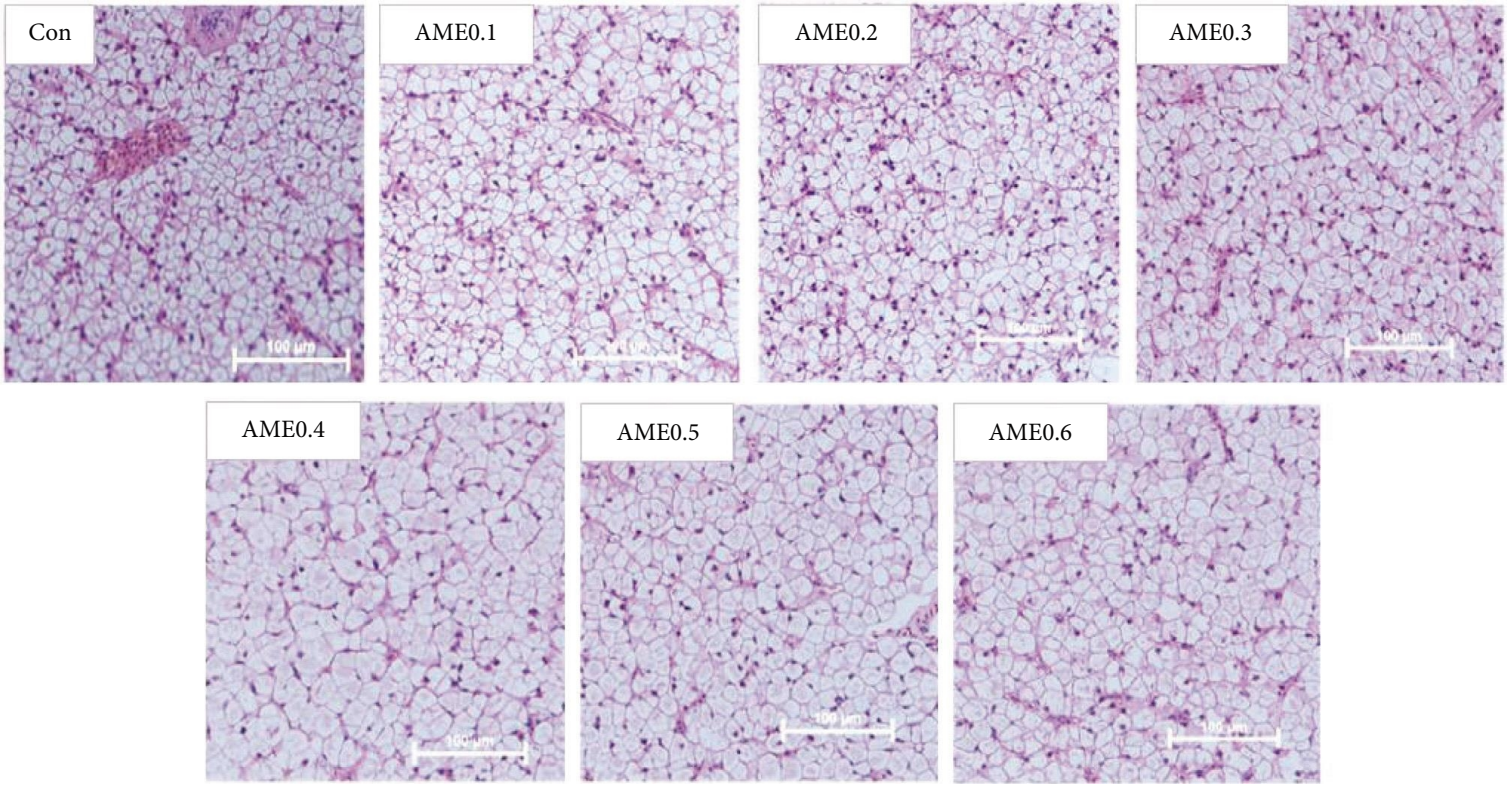
Effects of AME supplementation of different levels on hepatic histology of *M. salmoides* (H & E staining). Scale bar: 100 *μ*m.

**Figure 3:**
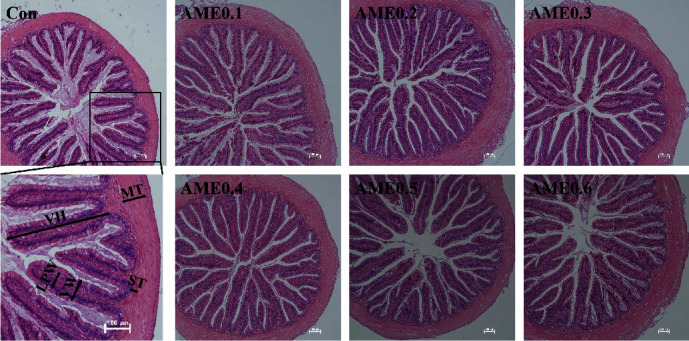
Effects of AME supplementation of different levels on intestinal histology of *M. salmoides* (H & E staining). VH, villi height; VW, villi width; LPW, lamina propria width; MT, muscular layer thickness; ST, submucosal layer thickness; scale bar: 100 *μ*m.

The hepatic and intestinal histology were presented in Table 6, Figures 2 and 3. The hepatocyte diameters of AME0.4 and AME0.6 groups were significantly higher than those of Con group (*P* < 0.05). In midgut, AME0.2 and AME0.3 groups had significantly longer villi height than Con group (*P* < 0.05). Fish of AME0.6 group had significantly higher lamina propria width than those of AME0.3 group (*P* < 0.05). The submucosal layer thickness was significantly larger in AME0.6 group than in all other groups except AME0.3 group (*P* < 0.05). AME treatment had no significant effects on midgut villi width or muscular layer thickness (*P* > 0.05).

### 3.6. Gene Expressions Related to Growth Performance

**Figure 4:**
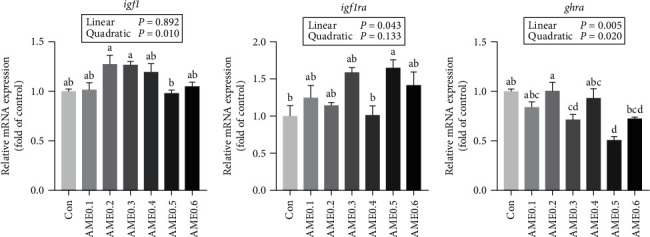
The mRNA expression level of growth-related genes of *M. salmoides* fed diets supplemented with different levels of AME. Values are means ± SEM of three replications. Different superscripts indicate significant differences (*P* < 0.05).

As presented in Figure 4, the expression level of *igf1* increased with incrementing dietary AME level up to 0.2%−0.3% and then decreased significantly (*P* < 0.05). The expression level of insulin-like growth factor 1a receptor (*igf1ra*) in AME0.5 group was significantly higher than those in Con group and AME0.4 group (*P* < 0.05). The transcription level of growth hormone receptor a (*ghra*) reached the highest in fish fed with 0.2% dietary AME, and then it showed a downward trend when dietary supplementation of AME kept leveling up.

### 3.7. Hepatic Gene Expressions Related to Inflammation and Apoptosis

**Figure 5:**
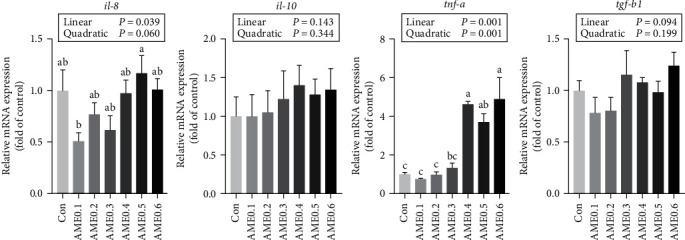
The mRNA expression level of inflammation-related genes of *M. salmoides* fed diets supplemented with different levels of AME. Values are means ± SEM of three replications. Different superscripts indicate significant differences (*P* < 0.05).

**Figure 6:**
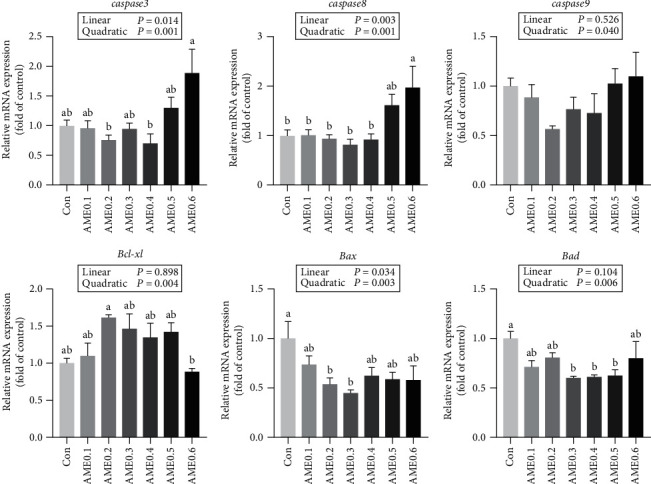
The mRNA expression level of apoptosis-related genes of *M. salmoides* fed diets supplemented with different levels of AME. Values are means ± SEM of three replications. Different superscripts indicate significant differences (*P* < 0.05).

As Figure 5 presented, AME0.1 group had a significantly lower mRNA expression level of *il-8* than AME0.5 group (*P* < 0.05). The transcription level of *tnf-a* was significantly upregulated when supplemented with more than 0.3% dietary AME (*P* < 0.05). No statistical differences were recorded in the expressions of interleukin 10 (*il-10*) and transforming growth factor *β*1 (*tgf-b1*) (*P* > 0.05). As shown in Figure 6, the expressions of *caspase3* and *caspase8* were both significantly enhanced in AME0.6 group (*P* < 0.05), while no statistical differences were shown in *caspase9* expressions (*P* > 0.05). The expression level of *Bcl-xl* in AME0.2 group was significantly higher than that in AME0.6 group (*P* < 0.05). Conversely, the transcription levels of *Bax* in AME0.2 and AME0.3 groups and *Bad* in AME0.3, AME0.4, and AME0.5 groups were significantly lower than those in Con group (*P* < 0.05).

### 3.8. Gene Expressions Related to Protein, Lipid, and Glucose Metabolism

**Figure 7:**
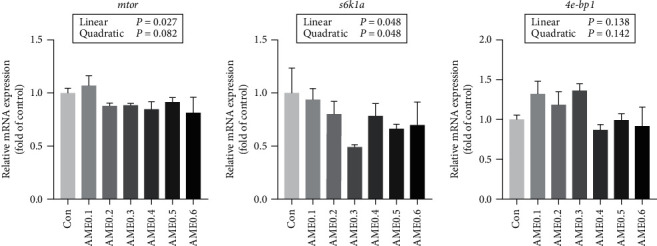
The mRNA expression level of protein synthesis-related genes of *M. salmoides* fed diets supplemented with different levels of AME. Values are means ± SEM of three replications. Different superscripts indicate significant differences (*P* < 0.05).

**Figure 8:**
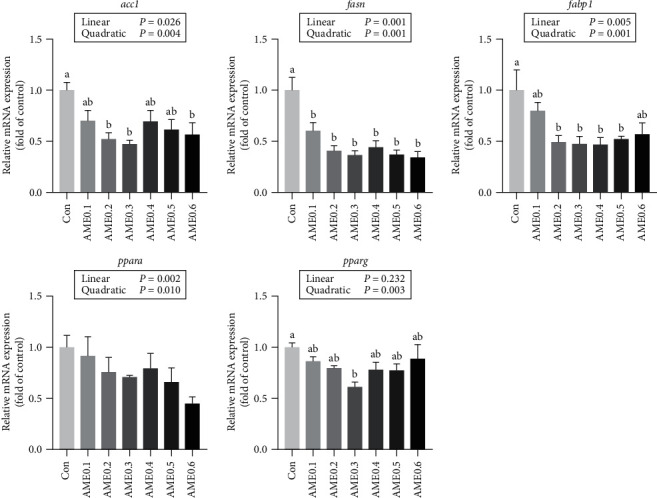
The mRNA expression level of lipid metabolism-related genes of *M. salmoides* fed diets supplemented with different levels of AME. Values are means ± SEM of three replications. Different superscripts indicate significant differences (*P* < 0.05).

**Figure 9:**
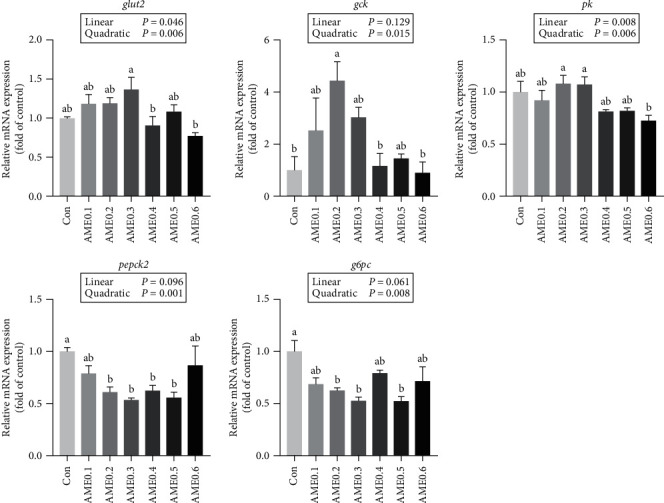
The mRNA expression level of glucose metabolism-related genes of *M. salmoides* fed diets supplemented with different levels of AME. Values are means ± SEM of three replications. Different superscripts indicate significant differences (*P* < 0.05).

For protein synthesis-related genes, the transcription levels of the mechanistic target of rapamycin (*mtor*), ribosomal protein S6 kinase b polypeptide 1a (*s6k1a*), and eukaryotic translation initiation factor 4E binding protein 1 (*4e-bp1*) all showed no significant differences (Figure 7). The hepatic expression levels of lipid metabolism-related genes are shown in Figure 8. AME supplementation significantly declined the transcription levels of both *acc1* and *fasn* (*P*  < 0.05), and the expression levels of *fabp1* were significantly reduced in all AME-supplemented groups except AME0.1 and AME0.6 groups (*P* < 0.05). The transcription level of peroxisome proliferator-activated receptor alpha (*ppara*) showed a downward trend as dietary AME increased (*P* > 0.05). The expression of peroxisome proliferator-activated receptor gamma (*pparg*) first declined and then elevated again as dietary AME level increased, with reaching the minimum at AME0.3 group (*P* < 0.05). As shown in Figure 9, the expression levels of glucose transporter 2 (*glut2*), *gck*, and *pk* followed the same fluctuating trend. They all increased at first and then decreased with the turning point existing at AME0.2 or AME0.3 groups. For *pepck2* and *g6pc*, Con group had significantly higher transcription levels than AME0.2, AME0.3, and AME0.5 groups (*P* < 0.05).

## 4. Discussion

The present study effectively elucidated the growth-promoting effects of AME supplementation on *M. salmoides*. The augmentation of FBW, FI, WG, and SGR, as well as the decline of FCR, were recorded in our rearing experiment, which was in accord with the findings in *L. calcarifer* [29], *S. maximus* [30], and *C. argus* [40]. However, AME or *Astragalus* polysaccharides treatment failed to optimize growth performance in hybrid grouper *Epinephelus lanceolatus*♂ × *Epinephelus fuscoguttatus*♀ [41] and sea cucumber *Apostichopus japonicus* [42], which could be possibly ascribed to the discrepancies in species, developmental stages, rearing environments, supplementation levels of active ingredients, and so on [29]. AME supplementation also positively affected morphological development in *M. salmoides* by significantly improving CF and downregulating VSI and HSI, whereas high levels of AME caused re-elevation of VSI and HSI, which might indicate that excessive AME hardly further benefit body development and even conversely initiate adverse effects. The remarkable enlargement of hepatocytes in fish of AME0.4 and AME0.6 groups further validated this speculation. Similar to the research on *L. calcarifer* [29]. The decline of FBW, WG, and SGR in AME0.5 and AME0.6 groups after reaching the highest value in AME0.4 group demonstrated the suppressive effects of excessive AME on growth performance, which could be possibly associated with the damage in liver tissue. It has been reported that antinutritional factors in plants, including phytic acid, protease inhibitors, tannins, and so on, negatively altered nutrient bioavailability in multiple organisms [43]. The residual antinutritional factors in the extraction products possibly imposed harmful effects on fish growth and liver integrity, and such effects became more and more prominent with the increase of AME supplementation level.

Meanwhile, the treatment of AME promoted crude protein contents in the whole body of *M. salmoides*. Similar results were obtained in researches on large yellow croaker *Larimichthys crocea* [44], suggesting an impressive function of AME in enhancing protein retention, which could be associated with the improvement of FCR and PER in AME groups. It was noteworthy that whole-body crude lipid contents in AME groups were lowered, following a trend completely opposite to that of body protein, while muscle crude lipid contents were remarkably upregulated by AME treatment, which displayed great capabilities of AME on gaining healthier fish body composition and higher nutritional value for human consumers. Plasma TG levels were reduced as well in AME groups compared with that of Con group, which was consistent with the results in researches on *L. calcarifer* [29]. A study on *E. lanceolatus*♂ × *E. fuscoguttatus*♀ revealed that AME decreased serum cholesterol contents while not altering TG levels [41], which just contradicted what we have illustrated in the present study, showing again the diverse regulatory effects of AME on different aquatic species. Besides lipid components, AME also functioned in lessening blood glucose contents, and similar findings were reported in bluegill sunfish *Lepomis macrochirus* [45]. Therefore, it could be concluded from the results above that AME or its active substances participated in the regulation of lipid and carbohydrate metabolism in *M. salmoides* and other fishes.

AST, ALT, AKP, and ACP are commonly selected as key indicators of liver diseases in clinical diagnosis [46]. Plasma AST and ALT elevation generally reflects the damage of hepatocytes [47]. AKP and ACP catalyze the removal of phosphate groups from molecules, including bacteria-originated lipopolysaccharides, and AKP mitigates inflammation, as well by mediating purinergic signaling [48, 49]. In the current study, plasma AST and ALT concentrations both declined in fish receiving AME treatment and simultaneously, AKP and ACP levels showed an increasing trend by proper amount of AME, which concomitantly indicated the enhancement of liver health and capability of defending against pathogenic threats. Multiple studies on AME and *Astragalus* polysaccharides have also described its effects in reducing AST and ALT in plasma or serum, including researches on *C. argus* [40], *L. macrochirus* [45], and crucian carp *Carassius auratus* [50]. Although serum AKP and ACP activities of Chinese mitten crab *Eriocheir sinensis* remained unchanged with the dietary intake of *Astragalus* polysaccharides [51], studies on *L. crocea* [44], *C. argus* [40], *L. vannamei* [34], and *A. japonicus* [42] all demonstrated the increase of AKP or ACP levels by supplementing *Astragalus* products, confirming the effects of AME on augmenting humoral innate immunity of aquatic animals. Studies on *C. argus* have also elucidated the same effects of flavonoids extracted from dandelion *Taraxacum mongolicum* and *Mongolia leek Allium mongolicum Regel* in reducing aminotransferases and enhancing phosphatases [33, 52], which also suggested the potential role of *Astragalus* flavonoids in improving nonspecific immunity.

The regulation of AME on antioxidant performance has also been investigated in our study. SOD alleviates cellular oxidative stress by converting superoxides to O_2_ and H_2_O_2_ [53]. The synthesized H_2_O_2_ is then broken down by CAT and GSH-PX into H_2_O and O_2_, effectively preventing abnormal accumulation of oxidants and counteracting the generation of reactive oxygen species (ROS) [54, 55]. Excessive ROS specifically targets carbon–carbon double bonds in polyunsaturated fatty acids mostly and leads to the production of MDA, a typical secondary product of lipid peroxidation for evaluating oxidative stress [56]. From our results, the decline of MDA contents, as well as the increase of CAT activities, clearly revealed the promoted antioxidative effects by AME treatment. Researches on *L. calcarifer* [29], *C. argus* [40], grass carp *Ctenopharyngodon idellus* [57] and Furong crucian carp *C. carpio L.♀* × *C. carpio var. Singguoensis♂* [58] reported as well the elevation of CAT activities, while decreased MDA contents were recorded in *L. crocea* [44] and *D. rerio* [28] treated with AME. The increased activities of SOD and GSH-PX were also elucidated in researches on the effects of AME or *Astragalus* polysaccharides on *S. maximus* [30], *L. macrochirus* [45], and Amur minnow *Phoxinus lagowskii* [59], and also enhanced antioxidant system in researches on effects of flavonoids on *C. argus* and *E. sinensis* [33, 52, 60], holistically emphasizing the beneficial effects of AME and *Astragalus* active components on protecting antioxidant capabilities.

Immunological performance correlates tightly with the integrity and normal functioning of the intestinal barrier. In our study, AME inclusion generally prolonged the midgut villi height of *M. salmoides*. Although the villi width showed no significant differences between Con group and other groups, it was slightly increased in AME groups. These collectively showed an increase in intestinal absorption area. Studies on *L. calcarifer* [29], *E. lanceolatus*♂ × *E. fuscoguttatus*♀ [41], *P. lagowskii* [59], and *C. carpio* [31] all reported the increase of intestinal villi height, presenting the effects of AME on potentiating gut digestion and absorption [61]. Apart from the influences on villi height, AME also notably altered the width of lamina propria and the thickness of the submucosal layer, suggesting the strengthening of gut immunity. It has been elaborated that intestinal mucosa serves as a powerful barrier against exterior pathogens [62], which relies highly on the functions of intestinal lamina propria. It regulates the adaptive immune system and protects epithelial barrier with the key involvement of immune cells, including macrophages and dendritic cells [63].

Cytokines are crucial factors in cell defense against pathogens, among which TNF-a has long been associated with pathogenesis. TNF-a functioning drives inflammatory responses and develops certain inflammatory diseases as well as tissue damage [64]. Il-8 is substantially produced as pro-inflammatory chemokine when inflammation commences and mediates the recruitment of neutrophils to the inflamed area [65], while Il-10 exerts anti-inflammatory activities in various cell types with inhibiting the synthesis of cytokines [66]. TGF-b1 effectively suppresses inflammatory reactions and inhibits T-cell activity, which prevents DNA damage and cancer development [67]. In our research, the inflammatory response was generally alleviated by lower levels of AME supplementation as indicated by the decrease of gene expression of *il-8*, while the increase of *tnf-a* expression suggested that higher levels of AME might conversely facilitate the progression of inflammation. Meanwhile, AME appeared to exert negligible effects on the expression of anti-inflammatory-related cytokines, since no significant differences were found in transcription levels of *il-10* and *tgf-b1*. *Astragalus* polysaccharides and flavonoids have been both ubiquitously proven in various aquatic species to enhance anti-inflammatory cytokines with suppressing pro-inflammatory factors, including *C. argus* [33, 40, 52], *L. calcarifer* [29], and *D. rerio* [28], indicating the great capability of AME in preventing aberrant inflammatory responses and thus maintaining liver health. It could be postulated that greater capabilities of eliminating oxidative stress as well as better tissue integrity, proved by hepatic antioxidant status and histological performance, made fish body less vulnerable to internal and external stresses, thereby reducing the level of inflammatory responses. However, AME overdose possibly induced immunological disorders caused by antinutritional factors, which led to inflammations with the involvement of pro-inflammatory factors, including TNF-a.

Caspase is well-known as an apoptosis-related family. Caspase3 is defined as executioner, while caspase8 and caspase9 are initiators, respectively, involved in extrinsic and intrinsic pathways of apoptosis [68]. Bcl-xl, Bax, and Bad all belong to the BCL-2 family, which is known for regulating apoptosis. Bcl-xl is normally anti-apoptotic, while both Bax and Bad are categorized as pro-apoptotic factors [69]. In our research, an increasing trend in *Bcl-xl* mRNA level, as well as a decreasing trend in *Bax* and *Bad* levels, manifested the alleviation of apoptosis by treatment of appropriate dosage of AME. Meanwhile, a significant rise in *caspase* transcription levels under the highest AME supplementing level revealed the negative effects of excessive AME in regulating cell death. Sun et al. [41] and Li et al. [58], respectively, reported the elevation of *caspase9* expression in head kidney of *E. lanceolatus*♂ × *E. fuscoguttatus*♀ and the decline of *caspase9* expression in liver and spleen of *C. carpio L*.♀ × *C. carpio var. Singguoensis*♂, while Du et al. [52] revealed the restraint of *Bax* and *caspase* expression in kidney of *C. argus*, displaying the regulatory effects of AME on cell apoptosis, with suggesting that such effects on caspase production varied a lot in different species and organs. Based on our results, AME-alleviated cell apoptosis by reducing oxidant generation and enhancing innate immunity, while cell death was increasingly induced when excessive AME was supplemented, which could be interpreted by the deterioration in tissue integrity of liver and gut.

The growth hormone-insulin-like growth factor 1 (GH-IGF1) pathway is a key regulator of growth and differentiation in vertebrates. GH could directly interact with its membrane-bound receptor GHRa to regulate growth and development. GH also induces the generation of IGF1 hormone and thus stimulates cell proliferation, with IGF1 receptor (IGF1R) primarily mediating the biological activities of IGF1 [70, 71]. In the present study, AME supplementation prominently increased the expressions of *igf1* and *igf1ra*, while exerting negligible influences on promoting the expression of *ghra*, suggesting that AME augmented the growth performance mainly by boosting the biological actions of IGF1 and IGF1R rather than targeting GHR for GH regulation.

Since both whole-body and muscle-proximate composition were significantly affected by AME treatment, the changes in mRNA expressions of energy metabolism-related genes were investigated in liver, one of the most important organs involved in metabolic regulation. The mTOR pathway perceives and responds to multiple internal and environmental changes by regulating cellular proliferation, with mTOR complex 1 playing a central role in stimulating protein synthesis [72]. Our results showed that the expression levels of *mtor* and its downstream genes (*s6k1a* and *4e-bp1*) remained relatively stable, suggesting that no extra protein was synthesized by receiving AME treatment. Hence, the increase in protein storage in the whole body and muscle could be possibly ascribed to the reduction in protein catabolism. Since no growth retardation was shown in AME groups, it could be assumed that lipids and carbohydrates played a key role in energy supply and maintenance of energy homeostasis, and changes in plasma TG and GLU contents preliminarily verified this postulation.

For lipid metabolism, ACC and FASN are both required for catalyzing the elongation of acetyl-CoA into fatty acids of 16 or 18 carbons, which are subsequently used for TG synthesis [73]. The decreased expression levels of *acc1* and *fasn* thus revealed the inhibiting effects of AME on *de novo* lipogenesis and TG generation. FABP1 was critically involved in fatty acid uptake and transport and was recently found to be an essential factor in cytoprotection against pathogens and oxidative stress [74]. PPARa mainly facilitates fatty acid oxidation in metabolically active sites and downregulates fatty acid levels [75], while PPARg is an important participant in the modulation of adipogenesis and lipid biosynthesis and also a regulator of fatty acid transport [76]. High affinity is revealed between FABP1 and PPARa, indicating their functional interactions [77], which is linked to the progression of fatty liver and nonalcoholic steatohepatitis [78]. In our study, the same decreasing trend in *fabp1* and *ppara* expression levels effectively proved the existence of PPARa-FABP1 axis regulation in *M. salmoides*. The reduction of *fabp1* expression in AME groups indicated less hepatic fatty acid influx and ameliorated hepatotoxic effects by AME treatment. The decrease of *ppara* and *pparg* expression levels suggested inhibited lipid oxidation and lipid synthesis in liver, meaning suppressed lipid metabolism and less lipid deposition in liver tissue. As lipid contents were increased in muscle by AME treatment, it could be postulated that AME triggered muscle-oriented lipid storage and prevented excessive hepatic fatty acid absorption, thereby relieved metabolic burden and helped prevent hepatic disorders, which showed high consistency with plasma immunological parameters like AST and ALT.

As for glucose metabolism, GLUT2 promotes the transport of glucose across the plasma membrane for cellular utilization [79]. GCK and PK are two rate-limiting enzymes that catalyze glycolysis. Specifically, GCK senses and facilitates the uptake of glucose by phosphorylation [80], while PK stimulates the synthesis of pyruvate for subsequent energy synthesis [81]. Gluconeogenesis, the opposing reaction of glycolysis, enables the synthesis of glucose from noncarbohydrate substrates, depending on the participation of PEPCK2 and G6PC [82, 83]. The increased expression levels of *glut2*, *gck*, and *pk* highly indicated the improved capabilities in utilizing glucose for energy generation by AME treatment, whereas the inhibition of *pepck2* and *g6pc* meant less glucose efflux, suggesting that glucose was well exploited for metabolic purposes and less was spared for storage in the form of glycogen. Therefore, it could be implied from the changes in energy metabolism-related parameters that AME positively modulated energy homeostasis by augmenting glucose uptake and metabolism as well as attenuating lipogenesis. Fatty-acid fueled less hepatic lipid synthesis and turned to relocation and deposition in muscle. Protein was less combusted for energy supply and more retained in fish body. In the whole, AME optimized the metabolic status in *M. salmoides* by exerting protein-sparing effects and preventing unnecessary retention of lipids and glucose. To sum up, AME significantly improved growth, immunological, and metabolic performance in *M. salmoides*, but not in a linear manner. Parameters, including SGR, WG, FCR, whole-body crude lipid contents, MDA, and ALT levels, were selected for determination of the optimal supplementation level of AME, and it is recommended to be 0.320%–0.429% of the diet based on second-degree polynomial regression analysis.

## 5. Conclusions

Our study elucidated that proper dietary supplementation of AME significantly potentiated the growth performance, antioxidant capacities, immunity, and tissue integrity of *M. salmoides*; AME treatment significantly alleviated inflammation response and apoptosis. Our study first verified the roles of AME in enhancing glucose utilization and inhibiting lipogenesis by altering the expressions of lipid and glucose metabolism-related genes, which resulted in the changes in whole-body and muscle-proximate compositions. The optimal AME supplementation level for *M. salmoides* was estimated to be 0.320%−0.429% of the diet based on polynomial regression analysis of SGR, WG, FCR, whole-body crude lipid, MDA, and ALT. Further investigation is required for clarifying the deeper mechanism of how AME affects whether different nutrients are used or stored in specific tissues and organs. Overall, these findings provide new insights into the utilization of AME as a great antioxidant, immunostimulant, and energy metabolism regulator for the culture of *M. salmoides* and probably some other aquatic species.

## Figures and Tables

**Table 1:** Ingredients and proximate composition of experiment diets (%, dry matter).

	Con	AME0.1	AME0.2	AME0.3	AME0.4	AME0.5	AME0.6
White fish meal^1^	42	42	42	42	42	42	42
Soybean meal^2^	16	16	16	16	16	16	16
Wheat flour^3^	11.7	11.7	11.7	11.7	11.7	11.7	11.7
Wheat gluten^4^	10	10	10	10	10	10	10
Beer yeast^5^	6	6	6	6	6	6	6
Krill meal^6^	2	2	2	2	2	2	2
Fish oil^7^	6	6	6	6	6	6	6
Soybean lecithin^8^	1	1	1	1	1	1	1
Multivitamin^9^	1	1	1	1	1	1	1
Multimineral^10^	1	1	1	1	1	1	1
Choline chloride (50%)^11^	0.5	0.5	0.5	0.5	0.5	0.5	0.5
Monocalcium phosphate^12^	1	1	1	1	1	1	1
Vitamin C^13^	0.2	0.2	0.2	0.2	0.2	0.2	0.2
Sodium alginate^14^	1	1	1	1	1	1	1
Cellulose microcrystalline^15^	0.6	0.5	0.4	0.3	0.2	0.1	0
*A. membranaceus* extract^16^	0	0.1	0.2	0.3	0.4	0.5	0.6
Total	100	100	100	100	100	100	100
Proximate composition (%)							
Moisture	9.33	9.55	9.47	9.37	9.48	9.06	9.14
Crude protein	49.51	49.18	49.30	49.80	49.10	50.12	49.91
Crude lipid	11.05	10.59	10.80	10.66	10.54	10.68	11.04
Ash	10.60	10.27	10.47	10.72	10.44	10.84	10.92
Gross energy (kJ/g)	17.31	17.05	17.16	17.22	17.01	17.30	17.40

^1^White fish meal: provided by Guangzhou Chengyi Industrial Group Co., Ltd., China, crude protein 63.58%, crude lipid 9.16%. ^2^Soybean meal: purchased from Yihai Kerry Jinlongyu Grain and Oil Food Co., Ltd., China, crude protein 46.39%, crude lipid 1.13%. ^3^Wheat flour: purchased from Hebei Jinshahe Noodle Industry Group Co., Ltd., China, crude protein 12.8%, crude lipid 2.8%, starch 62.5%. ^4^Wheat gluten: purchased from Henan Zaohua Grain and Oil Co., Ltd., China, crude protein 78.0%. ^5^Beer yeast: provided by Guangzhou Chengyi Industrial Group Co., Ltd., China, crude protein 41.91%. ^6^Krill meal: provided by Gongling Industrial (Shenzhen) Co., Ltd., China, crude protein 58.0%, crude lipid 12.0%. ^7^Fish oil: provided by Guangzhou Chengyi Industrial Group Co., Ltd., China. ^8^Soybean lecithin: provided by Guangzhou Chengyi Industrial Group Co., Ltd., China. ^9^Multivitamin (kg^−1^ diet): vitamin B1, 30 mg; vitamin B2, 60 mg; vitamin B6, 20 mg; nicotinic acid, 200 mg; calcium pantothenate, 100 mg; inositol, 100 mg; biotin, 2.5 mg; folic acid, 10 mg; vitamin B12, 0.1 mg; vitamin K3, 40 mg; vitamin A, 10,000 IU; vitamin D3, 2,000 IU; vitamin E, 160 IU. ^10^Multimineral (kg^−1^ diet): MgSO_4_·7H_2_O, 1,090 mg; KH_2_PO_4_, 932 mg; NaH_2_PO_4_·2H_2_O, 432 mg; FeC_6_H_5_O_7_·5H_2_O, 181 mg; ZnCl_2_, 80 mg; CuSO_4_·5H_2_O, 63 mg; AlCl_3_·6H_2_O, 51 mg; MnSO_4_·H_2_O, 31 mg; KI, 28 mg; CoCl_2_·6H_2_O, 6 mg; Na_2_SeO_3_·H_2_O, 0.8 mg. ^11^Choline chloride: provided by Guangzhou Chengyi Industrial Group Co., Ltd., China. ^12^Monocalcium phosphate: provided by Guangzhou Chengyi Industrial Group Co., Ltd., China. ^13^Vitamin C: provided by Guangzhou Chengyi Industrial Group Co., Ltd., China. ^14^Sodium alginate: purchased from Nanjing Duly Biotechnology Co., Ltd., China. ^15^Cellulose microcrystalline: purchased from Shanghai Acmec Biochemical Technology Co., Ltd., China. ^16^*A. membranaceus* extract: provided by Beijing Centre Biology Co Ltd., China.

**Table 2:** Growth performance, feed efficiency, and morphological parameters of *M. salmoides* fed diets supplemented with different levels of AME.

	Con	AME0.1	AME0.2	AME0.3	AME0.4	AME0.5	AME0.6	Regression	
								Linear	Quadratic
IBW^a^ (g)	10.12 ± 0.04	10.14 ± 0.01	10.16 ± 0.03	10.08 ± 0.03	10.05 ± 0.09	10.02 ± 0.02	10.07 ± 0.03	0.049	0.149
FBW^b^ (g)	58.64 ± 1.28^b^	59.47 ± 0.76^b^	62.49 ± 1.10^ab^	62.39 ± 2.07^ab^	64.86 ± 1.22^a^	60.16 ± 0.32^ab^	60.06 ± 0.88^ab^	0.402	0.007
SR^c^ (%)	96.67 ± 1.67	100.00 ± 0.00	100.00 ± 0.00	98.33 ± 1.67	98.33 ± 1.67	100.00 ± 0.00	100.00 ± 0.00	0.124	0.283
FI^d^ (g fish^−1^)	46.77 ± 0.74^bc^	47.37 ± 0.52^abc^	48.95 ± 0.53^ab^	49.88 ± 0.55^ab^	50.73 ± 0.67^a^	45.31 ± 0.32^c^	46.71 ± 0.99^bc^	0.682	0.006
WG^e^ (%)	478.14 ± 9.83^b^	486.71 ± 8.17^b^	514.97 ± 8.88^ab^	519.42 ± 19.04^ab^	550.07 ± 9.11^a^	501.21 ± 2.59^b^	496.47 ± 7.45^b^	0.181	0.002
SGR^f^ (% day^−1^)	3.13 ± 0.03^b^	3.16 ± 0.02^b^	3.24 ± 0.03^ab^	3.25 ± 0.06^ab^	3.34 ± 0.03^a^	3.20 ± 0.01^b^	3.19 ± 0.02^b^	0.170	0.001
FCR^g^	0.98 ± 0.01^a^	0.94 ± 0.01^ab^	0.93 ± 0.01^ab^	0.93 ± 0.02^ab^	0.92 ± 0.02^ab^	0.90 ± 0.01^b^	0.93 ± 0.01^ab^	0.009	0.003
PER^h^	2.06 ± 0.03^b^	2.14 ± 0.02^ab^	2.18 ± 0.01^ab^	2.15 ± 0.05^ab^	2.21 ± 0.05^a^	2.22 ± 0.02^a^	2.14 ± 0.01^ab^	0.039	0.004
CF^i^	2.02 ± 0.03^c^	2.17 ± 0.04^bc^	2.22 ± 0.03^ab^	2.22 ± 0.05^ab^	2.18 ± 0.04^bc^	2.32 ± 0.02^ab^	2.34 ± 0.03^a^	0.001	0.001
VSI^j^	8.79 ± 0.22^ab^	8.64 ± 0.14^ab^	8.20 ± 0.10^b^	8.44 ± 0.08^ab^	8.67 ± 0.11^ab^	8.99 ± 0.08^a^	8.71 ± 0.13^ab^	0.419	0.080
HSI^k^	2.48 ± 0.17^abc^	2.39 ± 0.14^abc^	2.10 ± 0.07^c^	2.21 ± 0.11^bc^	2.36 ± 0.00^abc^	2.76 ± 0.06^a^	2.64 ± 0.08^ab^	0.071	0.006

^a^Intial body weight (IBW, g) = initial total body weight/initial number of fish; ^b^Final body weight (FBW, g) = final total body weight/final number of fish; ^c^Survival rate (SR, %) = 100 × final number of fish/initial number of fish; ^d^Feed intake (FI, g fish^−1^) = 100 × total feed intake/final number of fish; ^e^Weight gain rate (WG, %) = 100 × (final body weight–initial body weight)/initial body weight; ^f^Specific growth rate (SGR, % day^−1^) = 100 × (ln (final body weight)–ln (initial body weight))/feeding days; ^g^Feed conversion ratio (FCR) = weight of feed consumed/weight gain; ^h^Protein efficiency rate (PER) = 100 × (final total body weight–initial total body weight)/(total feed intake × protein proportion in feed); ^i^Condition factor (CF, g cm^−3^) = 100 × body weight/body length^3^; ^j^Viscerosomatic index (VSI, %) = 100 × viscera weight/whole body weight; ^k^Hepatosomatic index (HSI, %) = 100 × liver weight/whole body weight. Values are means ± SEM of three replications. Means in the same row with different superscripts are significantly different (*P* < 0.05).

**Table 3:** Whole-body and muscle proximate composition of *M. salmoides* fed diets supplemented with different levels of AME.

	Con	AME0.1	AME0.2	AME0.3	AME0.4	AME0.5	AME0.6	Regression	
								Linear	Quadratic
Whole-body									
Moisture (%)	70.18 ± 0.13	70.31 ± 0.23	70.78 ± 0.05	70.92 ± 0.18	70.47 ± 0.24	70.87 ± 0.11	70.62 ± 0.20	0.047	0.030
Crude protein (%)	16.41 ± 0.03^b^	16.83 ± 0.16^ab^	17.05 ± 0.03^a^	16.60 ± 0.19^ab^	16.74 ± 0.13^ab^	16.28 ± 0.03^b^	16.49 ± 0.04^ab^	0.192	0.043
Crude lipid (%)	8.29 ± 0.14^a^	7.68 ± 0.07^ab^	7.41 ± 0.19^b^	7.29 ± 0.20^b^	7.75 ± 0.07^ab^	7.80 ± 0.09^ab^	7.83 ± 0.08^ab^	0.571	0.002
Ash (%)	3.81 ± 0.02^b^	3.92 ± 0.03^ab^	3.94 ± 0.06^ab^	3.99 ± 0.04^ab^	3.85 ± 0.06^ab^	3.91 ± 0.07^ab^	4.06 ± 0.05^a^	0.040	0.128
Muscle									
Moisture (%)	76.88 ± 0.03	76.71 ± 0.10	76.64 ± 0.02	76.47 ± 0.12	76.46 ± 0.11	76.49 ± 0.12	76.43 ± 0.04	0.001	0.001
Crude protein (%)	20.27 ± 0.11^ab^	19.95 ± 0.13^b^	20.23 ± 0.06^ab^	20.44 ± 0.14^a^	20.30 ± 0.02^ab^	20.19 ± 0.07^ab^	20.46 ± 0.11^a^	0.097	0.260
Crude lipid (%)	0.82 ± 0.09^b^	1.29 ± 0.13^a^	1.22 ± 0.03^a^	1.09 ± 0.02^ab^	1.23 ± 0.08^a^	1.24 ± 0.04^a^	1.19 ± 0.01^ab^	0.064	0.031
Ash (%)	1.50 ± 0.03	1.46 ± 0.06	1.36 ± 0.01	1.36 ± 0.01	1.42 ± 0.02	1.46 ± 0.03	1.49 ± 0.03	0.890	0.002

Values are means ± SEM of three replications. Means in the same row with different superscripts are significantly different (*P* < 0.05).

**Table 4:** Hepatic antioxidant abilities of *M. salmoides* fed diets supplemented with different levels of AME.

	Con	AME0.1	AME0.2	AME0.3	AME0.4	AME0.5	AME0.6	Regression	
								Linear	Quadratic
MDA^a^ (nmol mgprot^−1)^	0.53 ± 0.02^a^	0.48 ± 0.07^ab^	0.34 ± 0.05^ab^	0.28 ± 0.06^b^	0.29 ± 0.02^b^	0.42 ± 0.02^ab^	0.53 ± 0.06^a^	0.510	0.001
SOD^b^ (U mgprot^−1)^	456.16 ± 29.90	453.36 ± 19.20	432.50 ± 31.41	424.93 ± 27.50	474.95 ± 51.63	448.85 ± 20.65	499.84 ± 38.73	0.331	0.297
CAT^c^ (U mgprot^−1)^	8.00 ± 0.60^b^	12.06 ± 1.10^a^	12.12 ± 0.98^a^	11.10 ± 0.49^ab^	10.79 ± 0.52^ab^	11.28 ± 0.55^ab^	11.07 ± 0.56^ab^	0.260	0.056
GSH-Px^d^ (U mgprot^−1)^	22.99 ± 0.26	20.64 ± 2.13	18.87 ± 1.69	18.71 ± 2.05	19.05 ± 2.10	18.78 ± 1.03	19.24 ± 1.15	0.113	0.093

Values are means ± SEM of three replications. Means in the same row with different superscripts are significantly different (*P* < 0.05). ^a^MDA, malondialdehyde; ^b^SOD, superoxide dismutase; ^c^CAT, catalase; ^d^GSH-Px, glutathione peroxidase.

**Table 5:** Plasma biological and immunological parameters of *M. salmoides* fed diets supplemented with different levels of AME.

	Con	AME0.1	AME0.2	AME0.3	AME0.4	AME0.5	AME0.6	Regression	
								Linear	Quadratic
AST^a^ (U L^−1)^	12.01 ± 1.24^a^	9.40 ± 1.76^ab^	10.80 ± 1.14^ab^	7.73 ± 1.48^ab^	6.56 ± 0.71^ab^	5.70 ± 0.99^b^	6.05 ± 0.96^b^	0.001	0.001
ALT^b^ (U L^−1)^	7.33 ± 1.22^a^	3.48 ± 0.84^ab^	4.87 ± 1.37^ab^	2.73 ± 0.55^ab^	2.62 ± 0.95^ab^	1.82 ± 0.40^b^	4.49 ± 1.49^ab^	0.047	0.007
AKP^c^ (U L^−1)^	602.14 ± 30.08	679.84 ± 55.25	721.37 ± 24.42	718.02 ± 17.84	716.23 ± 45.42	695.92 ± 25.40	701.94 ± 21.85	0.083	0.021
ACP^d^ (U L^−1)^	651.73 ± 27.70	718.98 ± 56.63	799.24 ± 52.94	746.38 ± 37.16	640.94 ± 43.76	603.58 ± 47.52	633.47 ± 29.09	0.109	0.046
TG^e^ (mmol L^−1)^	5.18 ± 0.52^a^	4.24 ± 0.47^ab^	4.07 ± 0.17^ab^	5.01 ± 0.18^a^	4.44 ± 0.08^ab^	4.59 ± 0.19^ab^	3.49 ± 0.27^b^	0.064	0.145
T-CHO^f^ (mmol L^−1)^	11.14 ± 0.28	10.82 ± 0.81	10.85 ± 0.96	11.30 ± 0.83	10.41 ± 0.37	11.42 ± 0.47	10.26 ± 0.21	0.557	0.792
GLU^g^ (mmol L^−1)^	10.42 ± 0.38^a^	7.67 ± 1.09^ab^	7.02 ± 0.97^ab^	5.88 ± 0.27^b^	5.59 ± 0.38^b^	5.31 ± 0.45^b^	6.50 ± 1.12^ab^	0.002	0.001

Values are means ± SEM of three replications. Means in the same row with different superscripts are significantly different (*P* < 0.05). ^a^AST, aspartate aminotransferase; ^b^ALT, alanine aminotransferase; ^c^AKP, alkaline phosphatase; ^d^ACP, acid phosphatase; ^e^TG, triglyceride; ^f^T-CHO, total cholesterol; ^g^GLU, glucose.

**Table 6:** Hepatic and intestinal histological parameters of *M. salmoides* fed diets supplemented with different levels of AME.

	Con	AME0.1	AME0.2	AME0.3	AME0.4	AME0.5	AME0.6	Regression	
								Linear	Quadratic
Hepatic cell diameter (*μ*m)	16.45 ± 0.45^c^	16.67 ± 0.94^bc^	16.63 ± 0.77^bc^	16.28 ± 0.64^c^	20.32 ± 1.09^a^	19.19 ± 0.70^abc^	20.16 ± 0.77^ab^	0.001	0.001
Midgut villi height (*μ*m)	517.01 ± 11.72^b^	566.03 ± 22.63^ab^	640.78 ± 22.72^a^	620.81 ± 15.12^a^	591.15 ± 11.70^ab^	609.22 ± 30.45^ab^	612.20 ± 24.65^ab^	0.031	0.007
Midgut villi width (*μ*m)	89.95 ± 1.27	93.46 ± 3.04	93.08 ± 0.43	92.37 ± 2.12	91.37 ± 1.31	92.46 ± 4.61	89.40 ± 3.21	0.687	0.484
lamina propria width (*μ*m)	49.45 ± 0.94^ab^	49.67 ± 1.37^ab^	47.00 ± 2.01^ab^	44.94 ± 1.24^b^	49.18 ± 0.78^ab^	49.77 ± 1.98^ab^	53.02 ± 2.71^a^	0.209	0.012
Muscular layer thickness (*μ*m)	123.76 ± 13.87	122.71 ± 6.78	126.60 ± 8.95	127.50 ± 3.24	140.39 ± 3.74	133.71 ± 8.57	143.00 ± 5.33	0.024	0.078
Sub-mucosal layer thickness (*μ*m)	24.53 ± 0.89^b^	24.13 ± 0.99^b^	23.10 ± 0.80^b^	24.79 ± 0.89^ab^	24.30 ± 1.39^b^	23.89 ± 1.11^b^	29.82 ± 1.54^a^	0.058	0.009

Values are means ± SEM of three replications. Means in the same row with different superscripts are significantly different (*P* < 0.05).

## Data Availability

Data will be made available from the corresponding author upon reasonable request.

## Supplementary Materials

Supplementary 1 The procedure of preparing the *Astragalus membranaceus* extract (AME) powder.

Supplementary 2 The sequence information of primer pairs used for quantitative real-time PCR.
