# Supplementary material for: Dietary Supplementation of Astragalus membranaceus Extract Affects Growth Performance, Antioxidant Capacity, Immune Response, and Energy Metabolism of Largemouth Bass (Micropterus salmoides)
Source: Aquac Nutr. 2024 Mar 1;2024:3893671. doi: 10.1155/2024/3893671 (PMC10923623; doi:10.1155/2024/3893671)
Supplement: Supplementary 1 — The procedure of preparing the Astragalus membranaceus extract (AME) powder. [file 3893671.f1.docx]

The AME powder was prepared as follows:

1 kg dried *A. membranaceus* was fully crushed and boiled in 2 L distilled water for 1 h for primary extraction, and same extraction was repeated twice. The total extract was then concentrated by boiling gently and filtered for supernatant. The concentrated extract was then dissolved in absolute ethanol and thoroughly mixed with cellulose. By evaporation at 50 ℃, the ethanol was removed and the active components were co-precipitated with cellulose. The solid-phase precipitate was obtained by centrifugation and lyophilized for 24 h. The AME powder was finally harvested by pulverizing the product and sieving by a 180 μm mesh.
