# Supplementary material for: Dietary Supplementation of Astragalus membranaceus Extract Affects Growth Performance, Antioxidant Capacity, Immune Response, and Energy Metabolism of Largemouth Bass (Micropterus salmoides)
Source: Aquac Nutr. 2024 Mar 1;2024:3893671. doi: 10.1155/2024/3893671 (PMC10923623; doi:10.1155/2024/3893671)
Supplement: Supplementary 2 — The sequence information of primer pairs used for quantitative real-time PCR. [file 3893671.f2.docx]

Table S1 Sequences of gene primers for quantitative real-time PCR

| Genes | Forward primers (5’ to 3’) | Reverse primers (5’ to 3’) | Product  length (bp) | Annealing temperature (℃) | Sources / Genbank Accession No. |
| --- | --- | --- | --- | --- | --- |
| *igf1* | TCAAGAGTGCGATGTGCTGT | ATTGGGGCCATAGCCTGTTG | 191 | 60 | XM_038738328.1 |
| *igf1ra* | TCCACCCAAACGGGAAGATG | GTACTCCGGGTTGACAGACG | 169 | 60 | XM_038706666.1 |
| *ghra* | GAGTCCAGGCAACTTCCACA | GCATCGAAGAAGCACTCCCT | 135 | 60 | XM_038704747.1 |
| *il-8* | CGTTGAACAGACTGGGAGAGATG | AGTGGGATGGCTTCATTATCTTGT | 112 | 60 | Yu et al. [84] |
| *il-10* | CGGCACAGAAATCCCAGAGC | CAGCAGGCTCACAAAATAAACATCT | 119 | 60 | Yu et al. [84] |
| *tnf-a* | CTTCGTCTACAGCCAGGCATCG | TTTGGCACACCGACCTCACC | 161 | 60 | Yu et al. [84] |
| *tgf-b1* | GCTCAAAGAGAGCGAGGATG | TCCTCTACCATTCGCAATCC | 118 | 60 | Yu et al. [84] |
| *caspase3* | GCTTCATTCGTCTGTGTTC | CGAAAAAGTGATGTGAGGTA | 98 | 60 | Yu et al. [84] |
| *caspase8* | GAGACAGACAGCAGACAACCA | TTCCATTTCAGCAAACACATC | 195 | 60 | Yu et al. [84] |
| *caspase9* | CTGGAATGCCTTCAGGAGACGGG | GGGAGGGGCAAGACAACAGGGTG | 125 | 60 | Yu et al. [84] |
| *Bcl-xl* | CATCCTCCTTGGCTCTGG | GGGTCTGTTTGCCTTTGG | 141 | 60 | XM_038695757.1 |
| *Bax* | TCTTCACTCAGTCCCACAAA | ATACCCTCCCAGCCACC | 236 | 60 | XM_038704178.1 |
| *Bad* | CACATTTCGGATGCCACTAT | TTCTGCTCTTCTGCGATTGA | 116 | 60 | XM_038730645.1 |
| *mtor* | GAGGAGCTTATCCGAGTGGC | CTTTGAGGGTCTGTGGTCCC | 160 | 60 | XM_038723321.1 |
| *s6k1a* | GCCCAAGAACACCTGTGAGT | TGCCACTACTGCTTGTGTCC | 138 | 60 | XM_038708507.1 |
| *4e-bp1* | GACCCTGTTCAGCACGACC | AGTTCTCCATTGTGGGCCTT | 174 | 60 | XM_038703877.1 |
| *acc1* | ATCCCTCTTTGCCACTGTTG | GAGGTGATGTTGCTCGCATA | **121** | **60** | **XM_038709727.1** |
| *fasn* | CAGCCCTTGACTCATTCCG | CGCAGACTACGACCCGACAG | **259** | **60** | **XM_038735140.1** |
| *fabp1* | GAACCTCAAGGAGAGCCAGAA | CACCGTCCACCGAGATAATAGT | 116 | 60 | Zhang et al. [85] |
| *ppara* | CCACCGCAATGGTCGATATG | TGCTGTTGATGGACTGGGAAA | 144 | 60 | Yu et al. [84] |
| *pparg* | CCTGTGAGGGCTGTAAGGGTTT | TTGTTGCGGGACTTCTTGTGA | 103 | 60 | Yu et al. [84] |
| *glut2* | TTCACAGCAGTTCTCGGGTC | TTCTGACAGGTCATTGGCCC | 132 | 60 | XM_038728860.1 |
| *gck* | ACATTGTGCGTCTGGTCTGT | TCTTGTGGAACCTGTCACGG | 183 | 60 | XM_038703172.1 |
| *pk* | CAAGACGAGGCTGACCTGAG | CTTGCCTGCTTTCCACCTTG | 157 | 60 | XM_038711316.1 |
| *pepck2* | GCAGATCATGTCCTTCGGCA | CTACGTAACGCTTCACCCCC | 164 | 60 | XM_038725393.1 |
| *g6pc* | TCAGGTCATGCTATGGGTGC | TGACTTGGTGTGGGAAGTGG | 196 | 60 | XM_038735544.1 |
| *ef-1a* | GGCTGGTATCTCCAAGAACG | GTCTCCAGCATGTTGTCWCC | **239** | **60** | **KT827794.1** |

Abbreviations: igf1, insulin-like growth factor 1; igf1ra, insulin-like growth factor 1a receptor; ghra, growth hormone receptor a; il-8, interleukin 8; il-10, interleukin 10; tnf-a, tumor necrosis factor alpha; tgf-b1, transforming growth factor beta 1; caspase 3, cysteinyl aspartate specific protease 3; caspase 8, cysteinyl aspartate specific protease 8; caspase 9, cysteinyl aspartate specific protease 9; Bcl-xl, B-cell lymphoma-xl; Bax, bcl-2 associated x protein; Bad, bcl-2-associated death protein; mtor, mechanistic target of rapamycin; s6k1a, ribosomal protein S6 kinase b polypeptide 1a; 4e-bp1, eukaryotic translation initiation factor 4E binding protein 1; acc1, acetyl-CoA carboxylase alpha; fasn, fatty acid synthase; fabp1, fatty acid binding protein 1; ppara, peroxisome proliferator-activated receptor alpha; pparg, peroxisome proliferator-activated receptor gamma; glut2, glucose transporter 2; gck, glucokinase; pk, pyruvate kinase; pepck2, phosphoenolpyruvate carboxykinase 2; g6pc, glucose-6-phosphatase catalytic subunit 1a; ef-1a, elongation factor 1 alpha.

**References**

84.Yu, L.L., Yu, H.H., Liang, X.F., Li, N., Wang, X., Li, F.H., Wu, X.F., Zheng, Y.H., Xue, M., Liang, X.F., 2018. Dietary butylated hydroxytoluene improves lipid metabolism, antioxidant and anti-apoptotic response of largemouth bass (*Micropterus salmoides*). Fish Shellfish Immunol. 72, 220–229.

85.Zhang, W., Tan, B., Liu, K., Dong, X., Yang, Q., Chi, S., Liu, H., Zhang, S., Wang, H., 2019. Effects of different dietary lipids on growth, body composition and lipid metabolism‐related enzymes and genes in juvenile largemouth bass, *Micropterus salmoides*. Aquac. Nutr. 25, 1318–1326.
